# Integrating Health Belief Model and Technology Acceptance Model: An Investigation of Health-Related Internet Use

**DOI:** 10.2196/jmir.3564

**Published:** 2015-02-19

**Authors:** Ashraf Sadat Ahadzadeh, Saeed Pahlevan Sharif, Fon Sim Ong, Kok Wei Khong

**Affiliations:** ^1^Universiti Putra MalaysiaFaculty of Modern Languages and CommunicationKuala LumpurMalaysia; ^2^Taylor’s University MalaysiaTaylor’s Business SchoolSubang JayaMalaysia; ^3^The University of Nottingham Malaysia CampusNottingham University Business SchoolSemniyeMalaysia

**Keywords:** health beliefs, Technology Acceptance Model, health-related Internet use behavior, mediation effect

## Abstract

**Background:**

Today, people use the Internet to satisfy health-related information and communication needs. In Malaysia, Internet use for health management has become increasingly significant due to the increase in the incidence of chronic diseases, in particular among urban women and their desire to stay healthy. Past studies adopted the Technology Acceptance Model (TAM) and Health Belief Model (HBM) independently to explain Internet use for health-related purposes. Although both the TAM and HBM have their own merits, independently they lack the ability to explain the cognition and the related mechanism in which individuals use the Internet for health purposes.

**Objective:**

This study aimed to examine the influence of perceived health risk and health consciousness on health-related Internet use based on the HBM. Drawing on the TAM, it also tested the mediating effects of perceived usefulness of the Internet for health information and attitude toward Internet use for health purposes for the relationship between health-related factors, namely perceived health risk and health consciousness on health-related Internet use.

**Methods:**

Data obtained for the current study were collected using purposive sampling; the sample consisted of women in Malaysia who had Internet access. The partial least squares structural equation modeling method was used to test the research hypotheses developed.

**Results:**

Perceived health risk (β=.135, *t*_1999_=2.676) and health consciousness (β=.447, *t*_1999_=9.168) had a positive influence on health-related Internet use. Moreover, perceived usefulness of the Internet and attitude toward Internet use for health-related purposes partially mediated the influence of health consciousness on health-related Internet use (β=.025, *t*_1999_=3.234), whereas the effect of perceived health risk on health-related Internet use was fully mediated by perceived usefulness of the Internet and attitude (β=.029, *t*_1999_=3.609). These results suggest the central role of perceived usefulness of the Internet and attitude toward Internet use for health purposes for women who were health conscious and who perceived their health to be at risk.

**Conclusions:**

The integrated model proposed and tested in this study shows that the HBM, when combined with the TAM, is able to predict Internet use for health purposes. For women who subjectively evaluate their health as vulnerable to diseases and are concerned about their health, cognition beliefs in and positive affective feelings about the Internet come into play in determining the use of health-related Internet use. Furthermore, this study shows that engaging in health-related Internet use is a proactive behavior rather than a reactive behavior, suggesting that TAM dimensions have a significant mediating role in Internet health management.

## Introduction

### Health-Related Internet Use

Millions of people throughout the world use the Internet and much of this activity is focused on health [1,2]. The Internet is frequently used for seeking health information and communicating for health-related purposes [3-5]. Information seeking refers to the “purposive seeking for information as a consequence of a need to satisfy some goal” [6]. Individuals seek information to fill gaps between what they know and what they need to know in various fields including health. Health information seeking takes place in an environment where different sources are available [7] and information seekers consciously select 1 or more sources to meet their informational need [8]. Among formal and informal health information sources, however, mass media play a vital role in the dissemination of information: the Internet is a key source for information. The Internet as the largest online medical library contains more than 100,000 health-related websites [9]. Internet-based dissemination of health-related information is often suggested as an optimal way to spread health information [10] because the Internet provides privacy, immediacy, faster and easy access to a wide variety of health information, and a variety of perspectives on health-related issues [11,12].

The Internet not only functions as a rich source of health information, but it also provides interactivity between professionals and health seekers through an electronic or communication tool to gain and convey health information [13]. The interactive features of the Internet, such as emailing, chatting, and discussion forums, provide users with the opportunity to leave their questions related to their health and to contact with others, to share and exchange their experiences about a disease, to ask for the best physicians in the field, and to get and give psychological, emotional, and spiritual support from support groups such as bulletin boards and chat rooms [4,5,14,15]. All these communication-based activities on the Internet are not so easily performed through other media forms such as newspapers, radio, or television [16]. Internet use helps people make key health care decisions by connecting with those who access health information, and interacting with health professionals and social support groups [17].

Such importance placed on the Internet as a health-seeking platform helps people maintain, promote, and manage their health. Past research shows that women are more likely to use the Internet for health-related purposes than men [1,2]. In Malaysia, the use of the Internet to manage health and to learn more about diseases has become increasingly important [18] due to the increase in the incidence of chronic diseases, in particular among urban women [19]. Malaysian women, like women in other parts of the world, live longer than men, but are more susceptible to chronic diseases that are preventable [19]. The Internet can be beneficial for empowering women to take responsibility for their own health, decreasing the incidence of illness, and enhancing well-being. This could possibly explain why women are the dominant Internet users in terms of health information seeking even though the number of male Internet users is higher than that of female users [20].

### Health-Related Internet Use From the Health Belief Model Perspective

Although an abundance of research can be found on Internet health care information-seeking behavior, a major focus of these studies tends to concentrate on understanding the use of the Internet for health information-seeking behavior based on the Health Belief Model (HBM). The HBM was initially developed to predict the behavioral reaction of individuals with acute or chronic diseases to the treatment they receive [21], but the model was later employed to predict more general health behavior [22,23]. The basic assumption of the HBM is that, in the absence of any symptoms, individuals will not take health or preventive measures unless that they are psychologically ready (eg, they feel vulnerable to a disease) [21]. The HBM suggests that belief in health risk predicts the likelihood of engaging in health behavior [21]. Perceived health risks consist of 2 dimensions: perceived susceptibility to disease and perceived severity of disease. *Perceived susceptibility to disease* refers to “beliefs about the likelihood of getting a disease or condition” [21]. *Perceived severity of disease*, on the other hand, is defined as “feelings about the seriousness of contracting an illness or of leaving it untreated include evaluations of both medical and clinical consequences (eg, death, disability, and pain) and possible social consequences (eg, the effect of the condition on work, family life, and social relations)” [21].

**Figure 1:**
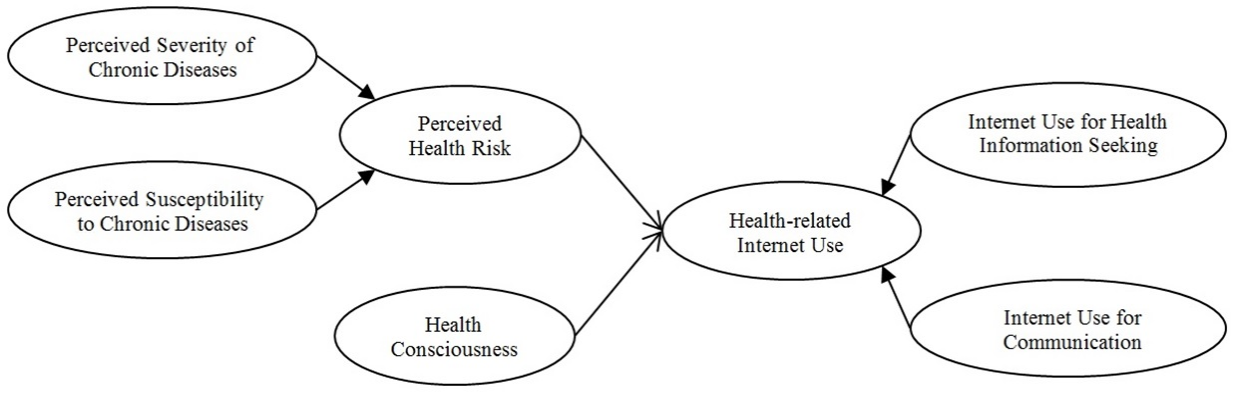
Hypothesized model based on the Health Belief Model.

Individuals with higher perceived health risk have greater motivation to change or adopt a health-oriented behavior, including adopting a preventive health behavior such as seeking information and using information and communication channels (eg, the Internet) to satisfy health-related information and communication needs [24-27] (Figure 1).

Results of past studies found that women tend to have a higher perceived health risk than men [28,29]. Moreover, perceived health risk is the most important and noticeable predictor in determining women’s health behavior adoption [30].

As well as perceived health risk, health consciousness is another dimension that influences health-seeking behavior. *Health consciousness* is defined as “the degree to which health concerns are integrated into a person’s daily activities” [31]. Health-conscious people are aware of and concerned about their wellness; therefore, they are motivated to improve and/or maintain their health.

Health consciousness is a predictor of the use of communication channels for health information seeking [32-34]. Health consciousness increases the amount of health-related information obtained from media sources such as television, radio programs, books, newspapers, magazines, advertising, and pamphlets about health [35]. The positive attitude toward the Internet has made it a primary health information source (Figure 1), in comparison to mass media (eg, television and radio), for learning about health-related issues [4,5,33] as previously discussed. In essence, the Internet has enabled individuals to be proactive in managing their health through seeking, exchanging, and communicating health-related information via the e-platforms.

### Health-Related Internet Use From the Technology Acceptance Model

Other studies that contribute toward the extant literature include those that are based on the Technology Acceptance Model (TAM) [36,37]. Although the HBM perspective explains health-related Internet use via the subjective assessment of an individual’s vulnerability to health risks and one’s consciousness toward health, the TAM views health-related Internet use behavior from the technology perspective (ie, the usefulness and ease of use of the Internet and one’s attitude toward Internet use) [38].

The TAM was developed to enable understanding of the use of technology [38] and is most commonly used for studying technology-related behavior such as the Internet and computer use in different contexts including health. The TAM has 3 dimensions: perceived usefulness, perceived ease of use, and attitude toward technology use. *Perceived usefulness* is defined “as the belief about using the technology that would bring benefits to the user,” whereas *perceived ease of use* refers to “the belief about using the technology that involves little effort” [36]. Perceived usefulness and perceived ease of use both affect attitude toward using the technology, which in turn influences behavioral intention to adopt the technology [36]. Attitude involves an individual’s belief about the consequences of performing a behavior (eg, technology use), whether it is good or bad, and the general evaluation influences an individual’s inclination to use or not to use a particular technology [39]. Attitude guides an individual’s behaviors by shaping perception [39].

**Figure 2:**
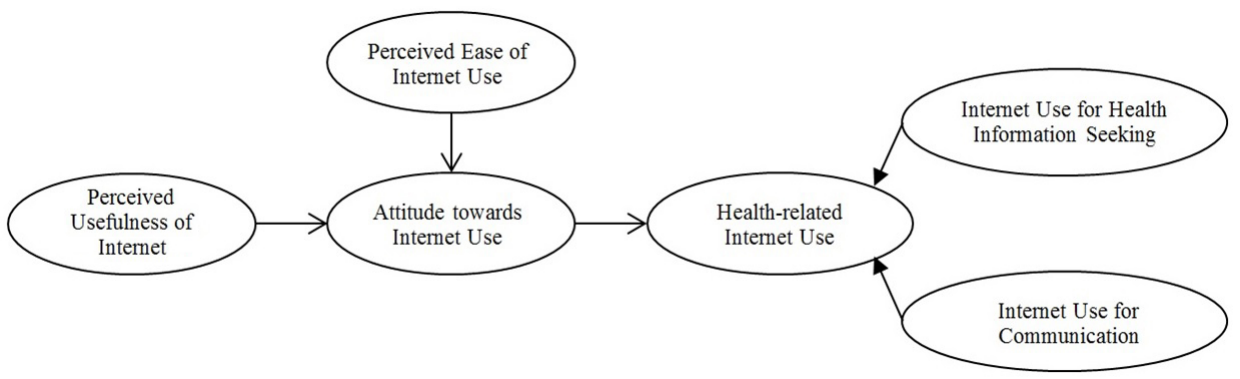
Hypothesized model based on Technology Acceptance Model.

Using the TAM framework, studies showed that perceived usefulness, perceived ease of use and attitude, positively influence behavioral intention to use health information technologies such as the Internet and mobile phones [36,40]. Furthermore, all studies that applied the TAM in the health care domain included behavioral intention to use health information technology, which is driven by the Internet [24,36,37] (Figure 2).

### Integrating the Health Belief Model and the Technology Acceptance Model

Although many past studies on Internet use for health-related purposes adopted the TAM or HBM, the use of these theories independently has not been able to explain fully Internet health-seeking behavior. The TAM has been used to predict an individual’s technology use; however, it is an inadequate model for health-related Web use because of its heavy dependence on 2 factors: perceived usefulness and perceived ease of use of technology [38]. The HBM attempts to explain the factors that influence health-related Internet use from purely the health perspective and it does not explain the mechanism or the process that lead to the behavior. Although the TAM has been widely used in explaining behavior that relates to technology [36,38,40], its effect on Internet use for health-related purposes could only be fully understood by incorporating dimensions of the HMB that explain individuals’ belief about health into the model. In short, there is a need to examine health-related Internet use from an integrated perspective that combines cognition, attitude, and behavior as well as the subjective evaluation of the psychological states of individuals regarding their perception of their health status.

**Figure 3:**
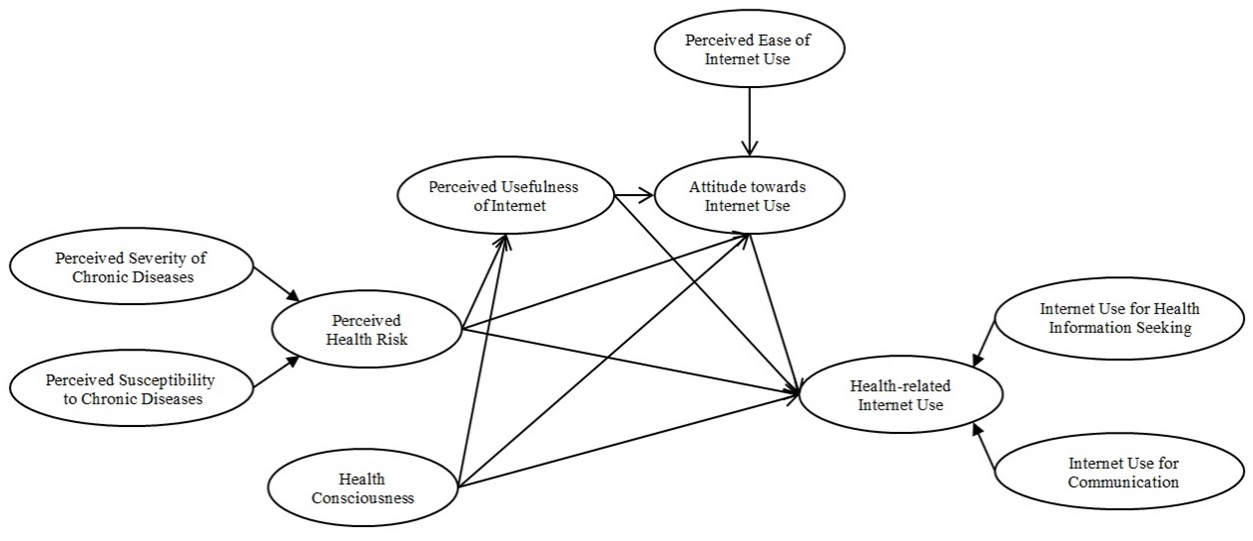
Integrated model based on the Health Belief Model and the Technology Acceptance Model.

By incorporating constructs of technology acceptance based on the TAM and perceived health risk and health consciousness as explained by the HBM, an integrated model of health-related Internet use behavior is proposed whereby perceived usefulness of the Internet and attitude toward the Internet for health purposes mediate the relationship between perceived health risks as well as health consciousness and health-related Internet use behavior (Figure 3). In this model, individuals who perceive their health to be at risk or are motivated to use the Internet when they believe that the Internet is useful for providing information on health and health management would be expected to have a positive attitude toward Internet use for health purposes. In other words, cognitive and affective beliefs toward the Internet become central to a person who perceives his/her health to be at risk or is conscious about health. Therefore, these individuals would have greater technology usage (ie, the Internet).

This study aimed to examine the influence of perceived health risk and health consciousness on health-related Internet use based on the HBM. The model developed for the purpose of this study incorporated the TAM to provide a better understanding of the process that affects the adoption of Internet use for health purposes. Based on the integrated model, this study set out to test the mediating effect of TAM constructs, perceived usefulness of the Internet, and attitude toward Internet use on the relationship between perceived health risk and health consciousness on Internet use for health purposes. Table 1 shows the 4 hypotheses developed for the purpose of this study based on the literature reviewed previously.

**Table 1:** Research hypotheses for explaining health-related Internet use drawing upon the Health Belief Model and the Technology Acceptance Model.

Research hypotheses	Path (causal effect)	Sources
H_1_: Perceived health risk toward chronic diseases consisted of perceived susceptibility to chronic diseases and perceived severity of chronic diseases has a positive effect on health-related Internet use	Perceived health risk → health-related Internet use	[21,25,26]
H_2_: Health consciousness has a positive effect on health-related Internet use	Health consciousness → health-related Internet use	[31-33]
H_3_: The effect of perceived health risk, consisted of perceived susceptibility to chronic diseases and perceived severity of chronic diseases, on health-related Internet use is mediated by perceived usefulness of the Internet, and attitude toward Internet use for health information and health management	Perceived health risk → perceived usefulness of the Internet → attitude toward Internet use → health-related Internet use	[36-38]
H_4_: The influence of health consciousness on health-related Internet use is mediated by perceived usefulness of the Internet, and attitude toward Internet use for health information and health management	Health consciousness → perceived usefulness of the Internet → attitude toward Internet use → health-related Internet use	[24,38]

## Methods

### Sample and Data Collection

The participants in this study consisted of Malaysian females living in the state of Selangor, the most urbanized state in Malaysia. Purposive sampling was used. Women who were Internet users were selected as the sample for the purpose of this study because past research found that they tend to be educated, married, and live in urban areas [15,41,42]. Furthermore, they tend to search for information regarding health [1,2,43]. Using the drop-and-collect method, a questionnaire was distributed to only those who expressed their willingness to be respondents. The purposive samples were sourced from women working in governmental institutions located in Selangor state through friends’ contacts.

Out of 380 questionnaires distributed, 330 completed questionnaires were obtained. From the 330 sets of questionnaires returned, 293 responses were usable after excluding cases that had not used the Internet for health-related purposes and cases with incomplete information.

As shown in Table 2, 127 of 293 respondents (43.0%) were in the 30 to 39 age group, 193 of 293 (66.5%) were married, 138 of 291 (47.5%) reported that they had a college or university degree, and 133 of 288 participants (46.0%) had an income in the range of 3000-5999 Malaysian Ringgit (RM) (US $882-$1764).

**Table 2:** Descriptive statistics of demographic characteristics of participants (N=293).

Characteristics		n (%)
**Age (years)**		
	20-29	110 (37.5)
	30-39	127 (43.0)
	40-49	43 (15.0)
	≥50	13 (4.5)
**Marital status**		
	Single	92 (31.5)
	Married	195 (66.5)
	Others	6 (2.0)
**Education level**		
	Primary school	18 (6.0)
	Secondary school	138 (47.5)
	College/university	135 (46.5)
**Household income (RM)**		
	1000-2999	114 (39.5)
	3000-5999	133 (46.2)
	6000-8999	39 (13.5)
	≥9000	2 (0.7)

### Measures

#### Perceived Health Risk

Perceived health risk contains 2 subdimensions: perceived susceptibility to chronic diseases and perceived severity of chronic diseases. Perceived susceptibility to chronic diseases was measured by 6 items adopted from Kim and Park [37] and Bryan et al [44]. Perceived severity to chronic diseases was measured by 4 items adopted from the Kim and Park study [37]. All items of these constructs were rated on a 5-point Likert-type scale (1=strongly disagree, 2=disagree, 3 =neutral, 4=agree, and 5=strongly agree) (Multimedia Appendix 1).

#### Health Consciousness

Participants’ health consciousness was measured by 11 items covering most facets of health consciousness adopted from Chen [45] and modified for this study. All items of these constructs were rated on a 5-point Likert-type scale (1=strongly disagree, 2=disagree, 3=neutral, 4=agree, and 5=strongly agree) (Multimedia Appendix 1).

#### Perceived Usefulness of the Internet for Health Information and Health Management

Items that measured perceived usefulness of the Internet for health information and health management were adopted from Davis [38]. All items of these constructs were rated on a 5-point Likert-type scale (1=strongly disagree, 2=disagree, 3=neutral, 4=agree, and 5=strongly agree) (Multimedia Appendix 1).

#### Perceived Ease of Internet Use

Perceived ease of Internet use was assessed by the 4 items developed by Davis [38]. All items of these constructs were rated on a 5-point Likert-type scale (1=strongly disagree, 2=disagree, 3=neutral, 4=agree, and 5=strongly agree) (Multimedia Appendix 1).

#### Attitude Toward Internet Use for Health Issues

Four items on attitudes toward Internet use for health information were adopted from the study by Wong et al [36]. All items of these constructs were rated on a 5-point Likert-type scale (1=strongly disagree, 2=disagree, 3=neutral, 4=agree, and 5=strongly agree) (Multimedia Appendix 1).

#### Health-Related Internet Use

Health-related Internet use had 2 subdimensions: Internet for seeking health and medical information and Internet use to communicate health-related issues. Internet use for health information seeking was measured by 11 items and Internet usage for communication on health-related issues was measured by 5 items adopted from past studies [5,37,46]. Respondents were asked to indicate how frequently they use the Internet for health and medical information and to communicate on health-related issues. All 16 items were rated on a 5-point Likert-type scale (5=always, 4=often, 3=sometimes, 2=rarely, and 1=never). A higher score indicated a higher frequency of Internet usage for health information seeking and communication for health-related issues (Multimedia Appendix 1).

## Results

**Figure 4:**
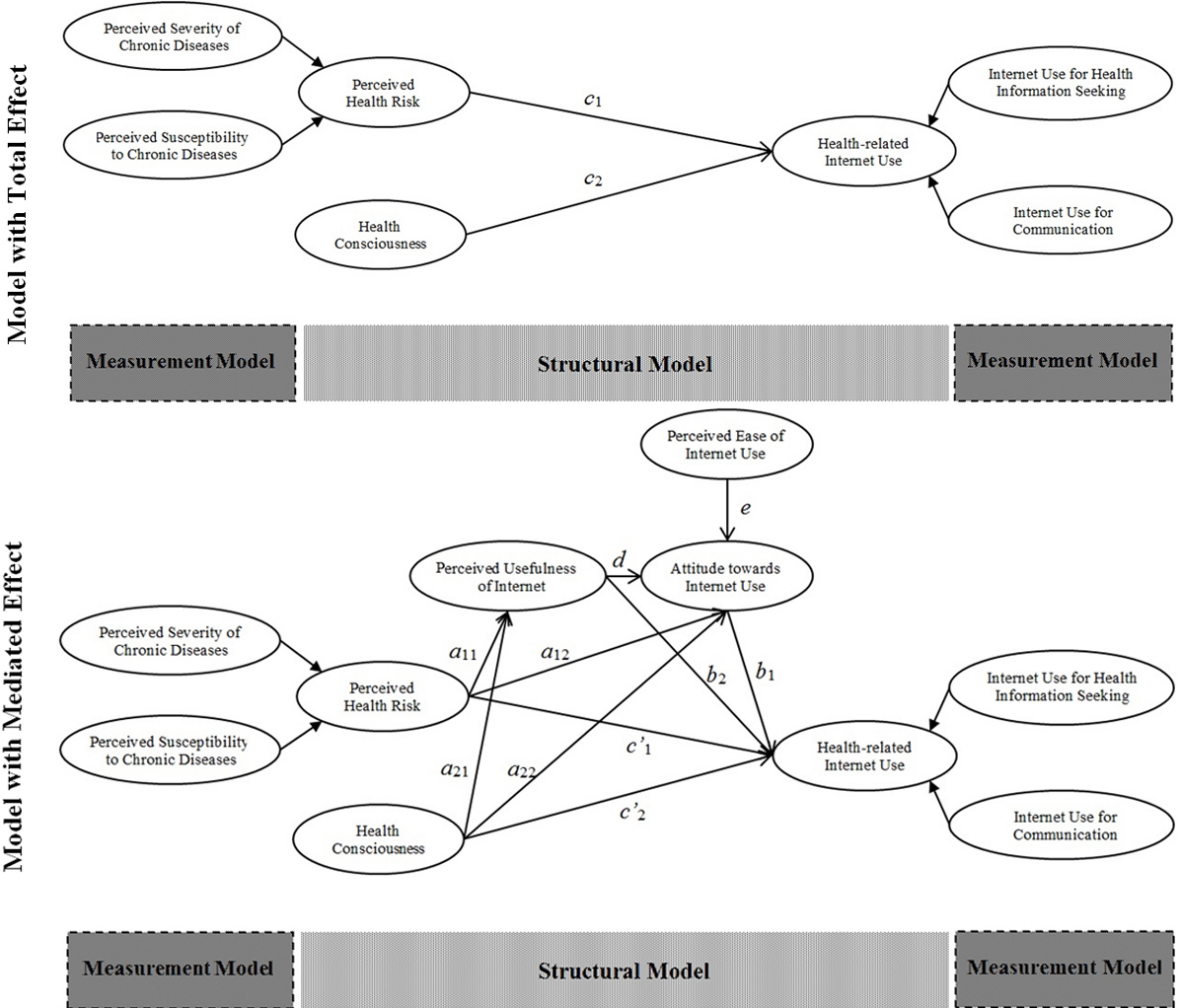
Structural research model.

We used the partial least squares structural equation modeling (PLS-SEM) method and SmartPLS software 2.0 [47] to estimate the structural model paths (Figure 4) and test the research hypotheses. PLS-SEM can cope with formative constructs and is appropriate for assessing relatively new measurement models. Both the constructs health-related Internet use (a second-order formative-formative construct) and perceived health risk (a second-order reflective-formative construct) justified the use of PLS-SEM for data analysis.

There are 3 different approaches to estimate parameters in models with second-order constructs: (1) the repeated indicator approach, (2) the 2-stage approach, and (3) the hybrid approach [48]. For the purpose of this study, a 2-stage approach was used. This is because the endogenous variable in the model of this study (health-related Internet use) is a formative second-order construct, which requires a 2-stage approach [48]. In the 2-stage method, first we specified the model with first-order constructs. Subsequently we estimated the latent variable scores of the first-order constructs and used these scores as indicators for the second-order constructs [48].

In order to discover the structure of reflective latent variables and to identify the underlying variance structure of a set of indicators, this research used exploratory factor analysis (EFA) [49]. Using oblique rotation, maximum likelihood factor extraction was performed on the 33 items of reflective constructs (refer to Table 2 for reflective constructs).

The Kaiser-Meyer-Olkin measure of sampling adequacy (0.816) and Bartlett’s test of sphericity results (*P*<.001) indicated the suitability of the data for factor analysis [50]. There were 54 (14.0%) nonredundant residuals with absolute values greater than .05 and the factors explain 63.71% of total variance. We excluded 5 items due to their low factor loadings and cross loadings over factors (ie, 1 item from perceived usefulness of the Internet, 1 item from perceived ease of Internet use, and 3 items from health consciousness). The details of the measurement properties of each reflective construct are reported in Table 3.

**Table 3:** Reflective constructs assessment.

Construct/measure		Factor loading^a^	Construct reliability	Average variance extracted	Maximum shared squared variance	Average shared square variance
**Perceived susceptibility to chronic diseases**			0.916	0.646	0.092	0.042
	I have a higher likelihood of getting chronic diseases	0.873				
	There is a great chance that I will be exposed to a chronic disease	0.808				
	I would say that I am the type of person who is likely to get chronic diseases	0.891				
	There is a person with chronic disease among my family members	0.759				
	I have a strong possibility of attack or deterioration of chronic disease due to improper daily habits (drinking, smoking, dietary habit, lack of exercise, etc)	0.707				
	It is most likely that I will catch chronic diseases in my lifetime	0.771				
**Perceived severity of chronic diseases**			0.900	0.694	0.022	0.011
	I am afraid of facing attack or deterioration of chronic diseases	0.756				
	If I face attack or deterioration of chronic disease, I will have difficulty with my work life (or domestic affairs)	0.807				
	If I face attack or deterioration of chronic disease, it will hinder my personal relationships	0.896				
	If I face attack or deterioration of chronic disease, I will be long haunted by resultant problems	0.865				
**Health consciousness**			0.925	0.608	0.228	0.140
	I have the impression that I sacrifice a lot for my health	0.791				
	I consider myself very health conscious	0.837				
	I think that I take health into account a lot in my life	0.876				
	I think it is important to know well how to stay healthy	0.883				
	My health is so valuable to me that I am prepared to sacrifice many things for it	0.766				
	I have the impression that other people pay more attention to their health than I do	0.767				
	I do not continually ask myself whether something is good for me	0.665				
	I often dwell on my health	0.610				
**Perceived ease of Internet use**			0.905	0.760	0.336	0.221
	My interaction with the Internet for health information is clear and understandable	0.857				
	I find the Internet for health information to be flexible to interact with	0.880				
	It is easy for me to become skillful at using the Internet for health information	0.878				
**Perceived usefulness of the Internet**			0.928	0.811	0.344	0.218
	Using the Internet is useful in managing my daily health	0.873				
	Using the Internet for health information is advantageous in better managing my health	0.937				
	Using the Internet for health information is beneficial to me	0.890				
**Attitude toward health-related Internet use**			0.933	0.777	0.344	0.303
	Using the Internet for health information and health management would be a good idea	0.894				
	Using the Internet for health information and health management would be a wise idea	0.872				
	I like the idea of using the Internet for health information and health management	0.895				
	Using the Internet for health information and health management would be a pleasant experience	0.865				

^a^The total variance explained by factors=63.713%. All factor loadings were more than 0.5 and significant (*P*<.05).

Subsequently, we assessed the construct reliability, convergent validity, and discriminant validity of reflective constructs [51]. Construct reliability greater than 0.7 is an acceptable reliability coefficient [51,52]. As shown in Table 3, the construct reliability of all reflective constructs varied from 0.900 to 0.933, which indicates good reliability. Then we assessed convergent and discriminant validity by estimating average variance extracted (AVE), maximum shared squared variance (MSV) and average shared square variance (ASV) [51,53]. For convergent validity, the results of this study show that the AVE of constructs exceeded 0.5 and construct reliability was greater than AVE, fulfilling the requirements of convergent validity [53].

To establish discriminant validity, both MSV and ASV should be less than the value of AVE. As shown in Table 2, MSV and ASV were less than AVE, indicating that there were no convergent and discriminant validity issues for the reflective constructs in this study.

In contrast to reflective constructs, indicators of formative constructs are not interchangeable and they do not necessarily have high intercorrelation [54]. In fact, high intercorrelation between indicators of formative constructs can increase the standard error, which results in instability of item coefficients [55]. Hence, instead of assessing reliability, convergent validity, and discriminant validity of formative constructs by conventional methods, we assessed them for collinearity issues [56,57].

In order to assess formative constructs, the collinearity issue was examined by computing correlation and the variance inflation factor (VIF). Table 4 shows maximum VIF and correlation between indicators of each formative construct. Because the maximum VIF for Internet usage for health information seeking and Internet usage to communicate for health indicators was less than 5, and indicators do not have high intercorrelation, this indicates an absence of a collinearity issue [58]. Further, to evaluate the contribution of formative indicators and their relevance, the factor weight of each indicator was assessed. As shown in Table 3, although only 3 indicators of Internet usage for health information seeking have significant weights, all outer loadings were greater than 0.5 (range 0.505-0.836). In addition, although Internet usage to communicate had 1 indicator with significant weight, factor loadings for all indicators were greater than 0.5 (range 0.655-0.931). Thus, all indicators of Internet usage for health information seeking and Internet usage to communicate made an absolute contribution to their respective constructs [58].

In the second stage of the 2-stage method, latent variable scores of perceived susceptibility to chronic disease and perceived severity of chronic disease as well as latent variable scores of Internet usage for health information seeking and Internet usage for communication were estimated and used to evaluate the formative second level of perceived health risks and health-related Internet use, respectively. The VIF of indicators of health-related Internet use and PHR was less than 5, which indicates an absence of collinearity issue. Moreover, the significant factor weights of perceived susceptibility to chronic disease, perceived severity of chronic disease, Internet usage for health information seeking, and Internet usage for communication show that they make a significant contribution to perceived health risks and health-related Internet use.

**Table 4:** Formative constructs assessment.

Construct/measure		Indicator weight	*t*_1999_	Indicator outer loading	Interitem correlation, mean (range)	Variance inflation factor, maximum
**Internet usage for medical and health information seeking**					0.536 (0.312-0.774)	30.665
	I use the Internet to get general health information	0.161	1.501	0.594		
	I use the Internet to get information on medicine/drugs	0.450	3.945	0.836		
	I use the Internet to be equipped with information before/after doctor’s appointment	–0.348	1.877	0.595		
	I use the Internet to get descriptions of various diseases	0.115	0.793	0.717		
	I use the Internet to get information on treatments/therapy/diagnosis	0.121	0.883	0.708		
	I use the Internet to get information on how to care for oneself	–0.201	1.468	0.567		
	I use the Internet to decide about how to treat an illness	0.444	3.011	0.803		
	I use the Internet to decide about whether or not to visit a doctor	0.097	0.735	0.735		
	I use the Internet to understand how to deal with an illness	0.111	0.610	0.643		
	I use the Internet to get information on hospitals/clinics/other health care facilities	0.257	2.112	0.717		
	I use the Internet to get information on health management (exercise, abstinence from drinking, smoking, diet, nutrition, stress, mental health, etc)	–0.002	0.015	0.505		
**Internet usage to communicate about health**					0.572 (0.441-0.685)	20.779
	I use the Internet to get online medical consultation from medical professionals	0.601	3.433	0.931		
	I use the Internet to interact with people with similar health conditions	0.280	1.462	0.833		
	I use the Internet to use mail to communicate with a doctor or a doctor’s office	–0.021	0.129	0.655		
	I use the Internet to share and exchange experiences about health and diseases	0.289	1.312	0.765		
**Health-related Internet use**					0.595	10.549
	Internet usage for medical and health information seeking	0.853	10.766	0.984		
	Internet usage to communicate for health	0.221	2.021	0.728		
**Perceived health risk**					0.005	10.000
	Perceived susceptibility to chronic diseases	0.946	14.430	0.948		
	Perceived severity of chronic diseases	0.319	1.967	0.324		

Next, in testing the hypotheses developed for this study, a bootstrapping resampling method with 2000 replications was performed [59]. Bootstrapping is a nonparametric approach that makes no distributional assumptions of variables and lets us estimate standard errors and confidence intervals and test the research hypotheses. In testing the mediation effect, a bootstrapping approach is more accurate and has higher statistical power than the approaches of Barron and Kenny [60], Sobel [61], and Taylor et al [62-64].

Standardized path coefficients, *t* value, and the percentile bootstrap 95% confidence interval of total, direct, and indirect effects on health-related Internet use are shown in Table 5.

**Table 5:** Direct, indirect, and total effects.^a^

Path			*R* ^*2*^	Q^2^	Standardized path coefficient, *β* (95% CI)	*t*_1999_(bootstrap)
**Total effect**						
	**Health-related Internet use**		.2395	.1531		
		Perceived health risk (c_1_)			.135^*^(.036, 234)	2.676
		Health consciousness (c_2_)			.447^***^(.351, .542)	9.168
**Direct effect**						
	**Perceived usefulness of the Internet**		.1821	.1460		
		Perceived health risk (a_11_)			.309^***^(.216, .401)	6.538
		Health consciousness (a_21_)			.269^***^(.165, .373)	5.063
	**Attitude toward Internet use**		.5284	.4074		
		Perceived usefulness of the Internet (d)			.334^***^(.224, .443)	5.955
		Perceived health risk (a_12_)			.063 (–.034, .160)	1.278
		Health consciousness (a_22_)			.270^***^(.167, .374)	5.118
		Perceived ease of Internet use (e)			.322^***^(.215, .429)	5.910
	**Health-related Internet use**		.3827	.2767		
		Attitude toward Internet use (b_1_)			.284^***^(.175, .392)	5.123
		Perceived usefulness of the Internet (b_2_)			.266^**^(.155, .377)	4.681
		Perceived health risk (c’_1_)			.019 (–.079, .117)	.383
		Health consciousness (c’_2_)			.211^***^(.107, .316)	3.958
**Indirect effect**						
	**Health-related Internet use**		.3827	.2767		
		Attitude toward Internet use, perceived usefulness of the Internet, perceived health risk (a_11_.d.b_1_)			.029^**^(.013, .045)	3.609
		Attitude toward Internet use, perceived usefulness of the Internet, health consciousness (a_21_.d.b_1_)			.025^*^(.010, .041)	3.234

^a^Arrows show the influence direction in the hypotheses. For example, perceived health risk influences (→) health-related Internet use.

* *P*<.05, ** *P*<.01, *** *P*<.001.

**Figure 5:**
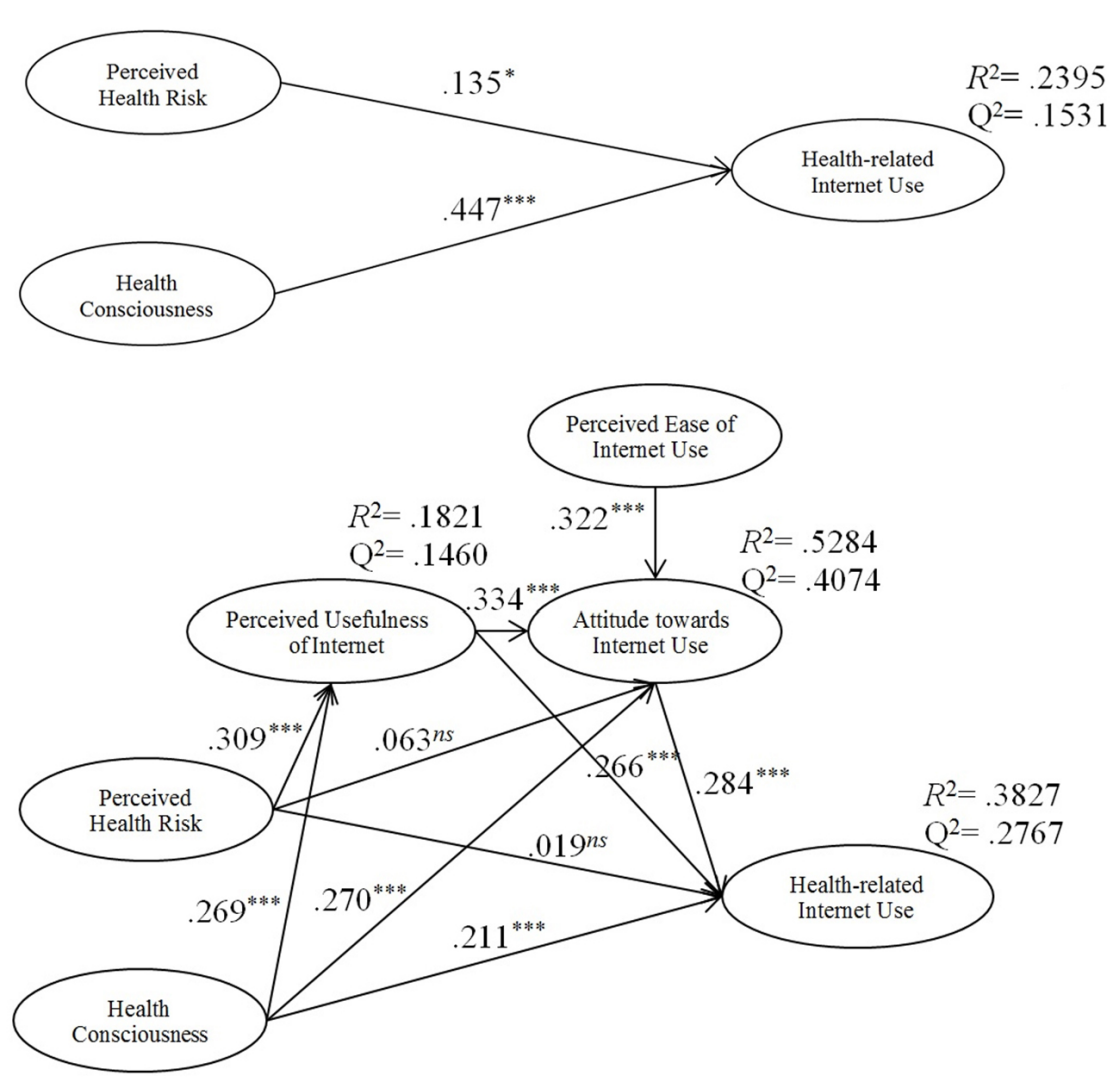
Path coefficients of the structural research model.

In testing hypotheses 1 and 2 on the effect of perceived health risk to chronic disease and health consciousness on health-related Internet use, the results show support for these 2 hypotheses as perceived health risk (β=.135, *t*_1999_=2.676) and health consciousness (β=.447, *t*_1999_=9.168) have significant positive influences on health-related Internet use (Figure 5).

Hypothesis 3 was developed to test the mediation role of perceived usefulness of the Internet and attitude in the relationship between perceived health risk and Internet use for health information seeking. Results showed that 8 of 10 direct effects described in the structural mediated effect model in Figure 4 were significant at the 95% confidence level or higher, whereas the direct effect of perceived health risk on attitude toward Internet use and health-related Internet use was not significant.

The indirect effect of perceived health risk on health-related Internet use through perceived usefulness of the Internet and attitude toward Internet use was significant at the 95% confidence level (β=.029, *t*_1999_=3.609). However, by controlling the mediators, the direct effect of perceived health risk on health-related Internet use was not significant and this indicated that perceived usefulness of the Internet and attitude toward Internet use fully mediated the effect of perceived health risk on health-related Internet use and hypothesis 3 was supported (Figure 5).

For hypothesis 4, the results showed that the indirect effect of health consciousness on health-related Internet use through perceived usefulness of the Internet and attitude toward Internet use was significant at the 95% confidence level (β=.025, *t*_1999_=3.234). Because the direct effect of health consciousness on health-related Internet use was significant (β=.211, *t*_1999_=3.958), perceived usefulness of the Internet and attitude toward Internet use partially mediated the effect of health consciousness on health-related Internet use, supporting hypothesis 4 (Figure 5).

The results showed support for all the hypotheses developed in the study. Further, the model explained 38.27% of the variance in health-related Internet use. To assess the predictive accuracy of endogenous variables, we used Stone-Geisser’s Q^2^ [65,66], which was implemented by a blindfolding procedure in SmartPLS 2.0. The results of predictive accuracy, shown in Table 4, indicated appropriate predictive power for all endogenous variables in the model (range 0.1460-0.4074) [67].

## Discussion

### Principal Findings

This study showed that there is a positive influence of perceived health risk and health consciousness on health-related Internet use, supporting hypotheses 1 and 2. It was also found that the effect of perceived health risk on health-related Internet use is fully mediated by perceived usefulness of the Internet and attitude toward Internet use for health information and health management as hypothesized in hypothesis 3. The study also supported that perceived usefulness of the Internet and attitude toward Internet use for health information and health management partially mediates the influence of health consciousness on health-related Internet use as proposed in hypothesis 4.

This study showed that perceived health risk positively affects health-related Internet use, confirming that perceived health risk is significant in influencing women’s Internet use for health-related purposes. This finding is consistent with Dillard et al’s study [26]. In addition, the results of the present study are in-line with Kim and Park’s study, which found that behavioral intention to use health information technology was influenced by perceived health risk [37]. However, the results of Kim and Park [37] showed a smaller impact of perceived health risk on intention to use health information technology (β=.016) than in this study (β=.135) One explanation for this could be related to the perceived health risk level of the participants, whereby the present study is based on urban women who tend to assess their health as being more at risk, whereas the sample of Kim and Park’s study consisted of both men and women [37].

The results of this study also showed that health consciousness has a significant positive effect on health-related Internet use, supporting the relevance of the HBM, which asserts that health consciousness contributes to health behavior adoption [31]. Additionally, it is consistent with prior research that revealed that health-conscious people engage more in health information-seeking behavior [32,33], prefer health information sources [68] and information oriented on the Internet [33], and tend to take part in both offline and online health communities [69].

The findings show that perceived usefulness of the Internet for health management and attitude toward Internet use for health-related purposes become central to women who perceive their health to be at risk and have the consciousness to seek information on health and health-related issues to manage their health and to stay healthy. Therefore, Internet use for health-related purposes is a process with perceived health risk and health consciousness as antecedents, but for this psychological orientation to translate into health-related Internet use behavior, perceived usefulness of the Internet and perceived ease of Internet use as well as attitude toward Internet use for health purposes provide the mechanism that explains health-related Internet use. In other words, for those who subjectively assess their health as susceptible to diseases and are concerned about their health, cognitive beliefs and positive affective feelings about the Internet come into play in the use of the Internet for health-related purposes.

Additionally, this integrated model shows that as health-related Internet use is predicted more by health consciousness than perceived health risk, it can be said that Internet usage for health purposes is a proactive health behavior driven by consciousness rather than a reactive health behavior. This result suggests that the Internet has become a necessary part of life for women who are health conscious and who prefer to be empowered by seeking health information online. Based on the findings of this study, the implications tend toward further promotion of Internet use for health purposes by individuals, health care service providers, and public policy makers. Knowing that health-related factors (ie, perceived health risk and health consciousness), technology-related cognitive beliefs (ie, perceived usefulness and perceived ease of use), and affective feelings toward Internet usage for health information positively influence Internet usage for searching health information, health care service providers could make greater use of the Internet to disseminate health-related information. Furthermore, health care providers can promote the use of online patient support systems or online self-care for a more seamless operation of their services. Individuals, especially women, would be motivated to seek information about health care by using the Internet, acting as opinion leaders in health and health-related issues for their family members and friends. Since the governments of all countries are keen to promote a healthy lifestyle, public policy makers could make use of the Internet to promote good health behavior, through women as the gatekeepers and as opinion leaders.

### Limitations

The present study has several limitations. First, the sample population focused only on working women living in urban areas. The sample was not representative of the Malaysian female population. Therefore, a more comprehensive future study is suggested to include both men and women with different ethnicities, age groups, household income levels, educational attainment levels, and place of residence for a more representative study. Second, apart from perceived health risk and health consciousness examined in this study, there are other health-related factors such as health locus of control, and health informational and decisional involvement that could be included in the deliberate reasoning process of health-related Internet use as moderator or exogenous constructs. Further, this study did not examine the influence of possible predictors of perceived ease of Internet use for health such as eHealth literacy. Therefore, we suggest that future studies could be devoted to examining the influence of these suggested constructs on health-related Internet use. Finally, based on the commonly known health-related activities that are most often performed on the Internet (namely health information seeking, communicating for health-related purposes, and purchasing drugs and health products), further studies could include purchase of drugs and other health care products as variables to enable better understanding of the use of the Internet for health maintenance activities.

### Conclusions

Although the present study supported past research that perceived health risk and health consciousness can operate as determinants of health-related Internet use as underpinned by HBM, the HBM model is insufficient to explain the mechanism for the adoption of the Internet for health purposes. By integrating HBM and TAM, results of this study provided the insight and an understanding that perceived usefulness of the Internet for health information and attitude toward Internet usage for health purposes act as mediators on the effect of health-related factors on health-related Internet use.

## Multimedia Appendix 1

Questionnaire.

## Abbreviations

ASV: average shared square variance
AVE: average variance extracted
HBM: Health Belief Model
MSV: maximum shared squared variance
TAM: Technology Acceptance Model
VIF: variance inflation factor
