# Supplementary material for: Integrating Health Belief Model and Technology Acceptance Model: An Investigation of Health-Related Internet Use
Source: J Med Internet Res. 2015 Feb 19;17(2):e45. doi: 10.2196/jmir.3564 (PMC4376166; doi:10.2196/jmir.3564)
Supplement: Supplementary file 1 [file jmir_v17i2e45_app1.pdf]

Questionnaires

Constructs	Items	Source(s)
**Perceived susceptibility to chronic diseases**	1-There is a great chance that I will be exposed to a chronic disease.<br>2-There is a person with chronic disease among my family members.<br>3-It is most likely that I will catch chronic diseases in my lifetime.<br>4-I have a higher likelihood of taking chronic diseases.<br>5-I have a strong possibility of attack or deterioration of chronic disease due to improper daily habits (drinking, smoking, dietary habit, lack of exercise, etc.).<br>6-I would say that I am the type of person who is likely to get chronic diseases.	Items 1-3 [1]<br>Items 4-6 [2]
**Perceived severity to chronic diseases**	1-I am afraid of facing attack or deterioration of chronic diseases.<br>2-If I face attack or deterioration of chronic disease, I will have difficulty with my work life (or domestic affairs).<br>3-If I face attack or deterioration of chronic disease, it will hinder my personal relationships.<br>4-If I face attack or deterioration of chronic disease, I will be long haunted by resultant problems.	[2]
**Health consciousness**	1-I have the impression that I sacrifice a lot for my health.<br>2-I consider myself very health conscious.<br>3-I think that I take health into account a lot in my life.<br>4-I think it is important to know well how to stay healthy.<br>5-My health is so valuable to me, that I am prepared to sacrifice many things for it.<br>6-I have the impression that other people pay more attention to their heath than I do. (R)<br>7-I do not continually ask myself whether something is good for me. (R)<br>8-I really don’t think often about whether everything I do is healthy. (R)<br>9-I don’t want to ask myself all the time, whether the things I eat are good for me. (R)<br>10-I often dwell on my health.<br>11-I am prepared to do many things to have good health.	[3]
**Perceived usefulness of the Internet**	1-Using the Internet for health information is useful in managing my daily health.<br>2-Using the Internet for health information is advantageous in better managing my health.<br>3-Using the Internet for health information is beneficial to me.<br>4-Using the Internet for health information is valuable to my healthcare.	[4]
**Perceived ease of Internet use**	1-Learning to use the Internet for searching health information was easy for me.<br>2-My interaction with the Internet for health information is clear and understandable.<br>3-I find the Internet for health information to be flexible to interact with.<br>4-It is easy for me to become skillful at using the Internet for health information.	[4]
**Attitude**	1-Using the Internet for seeking health information and health management would be a good idea.<br>2-Using the Internet for seeking health information would be a wise idea.<br>3-I like the idea of using the Internet for seeking health information and health management.<br>4-Using the Internet for seeking health information and health management would be a pleasant experience.	[5]
**Internet use for health information**	1-I use the Internet to get general health information.<br>2-I use the Internet to get description of various diseases.	Items 1-7 [6]

seeking	3-I use the Internet to get information on medicine/drug.<br>4-I use the Internet to be equipped with information before/after doctor's appointment.<br>5-I use the Internet to decide about whether or not to visit a doctor.<br>6-I use the Internet to decide about how to treat an illness.<br>7-I use the Internet to understand how to deal with an illness.<br>8-I use the Internet to get information on treatments/therapy/ diagnosis.<br>9-I use the Internet to get information on hospitals/clinics/other healthcare facilities.<br>10-I use the Internet to get information on health management (exercise, abstinence from drinking, smoking, diet, nutrition, stress, mental health, etc.).<br>11-I use the Internet to get information on how to care for oneself.	Items 8 & 9 [2]<br>Items 10 &11 [7]
**Internet use to communicate about health**	1-I use the Internet to get social support from other users via bulletin board, chat room, or conference.<br>2-I use the Internet to get online medical consultation from medical professionals.<br>3-I use the Internet to interact with people with similar health conditions.<br>4-I use the Internet to use mail to communicate with a doctor or a doctor's office.<br>5-I use the Internet to share and exchange experiences about health and diseases	Item 1 & 2 [6]<br>Items 3-5 [7]

References

1. Bryan AD, Aiken LS, West SG. Young women's condom use: The influence of acceptance of sexuality, control over the sexual encounter, and perceived susceptibility to common STDs. Health Psychology. 1997;16(5):468-479. PMID: 9302544

2. Kim J, Park H-A. Development of a Health Information Technology Acceptance Model Using Consumers' Health Behavior Intention. Journal of Medical Internet Research. 2012;14(5):e133. Doi: 10.2196/jmir.2143 PMID: 23026508

3. Chen MF. The joint moderating effect of health consciousness and healthy lifestyle on consumers' willingness to use functional foods in Taiwan. Appetite. 2011;57(1):253-262. Doi: 10.1016/j.appet.2011.05.305. PMID: 21609743

4. Davis F. Perceived usefulness, perceived ease of use, and user acceptance of information technology. Management Information Systems Quarterly. 1989;13(3):319-340.

5. Wong CK, Yeung DY, Ho HC, Tse K-P, Lam C-Y. Chinese Older Adults' Internet Use for Health Information. Journal of Applied Gerontology. 2012;32(8):1-20. Doi: 10.1177/0733464812463430. PMID: 24717738

6. Yoo EY, Robbins LS. Understanding middle-aged women's health information seeking on the web: A theoretical approach. Journal of the American Society for Information Science and Technology. 2008;59(4):577-590. Doi: 10.1002/asi.20766

7. Hale TM, Cotten SR, Drentea P, Goldner M. Rural-urban differences in general and health-related internet use. American Behavioral Scientist. 2010;53(9):1304-1325. Doi: 10.1177/0002764210361685.
